# Reactive soft tissue growth: a case report of onset post-extraction of natal tooth

**DOI:** 10.3389/fdmed.2026.1813485

**Published:** 2026-05-28

**Authors:** Kelsey O’Hagan-Wong, Theo Ratelle, Bridget Yichen Wu, Deepika Chugh, Michelle Yuchen Huang

**Affiliations:** 1 Holland Bloorview Kids Rehabilitation Hospital, Toronto, ON, Canada; 2 Bloorkids Dental, Toronto, ON, Canada; 3 University of Toronto Faculty of Dentistry, Toronto, ON, Canada

**Keywords:** natal tooth, pediatric dentistry, peripheral ossifying fibroma (POF), post extraction complication, soft tissue lesion

## Abstract

Natal teeth occur in approximately 1 in 2,000–3,000 live births and may cause oral soft tissue trauma such as Riga-Fede disease. This report describes a late-preterm female neonate with two mandibular incisor natal teeth, which were extracted. Following extraction, an erythematous gingival swelling developed at the site, which enlarged over five months into a firm, pink, pedunculated mass. Excision was performed under local anesthesia using a diode laser. Histopathology revealed a peripheral ossifying fibroma (POF)—a reactive inflammatory hyperplastic lesion that is exceedingly rare in infants. Healing was uneventful, with no recurrence. This case highlights the importance of monitoring extraction sites for delayed reactive lesions in neonates, even after resolution of the initial trauma, and emphasizes the value of histopathologic evaluation to guide management.

## Introduction

Natal teeth—teeth present at birth—are rare and most often involve the mandibular central incisors (1). Complications include feeding difficulty, aspiration risk, and oral soft tissue injury, particularly Riga-Fede disease (2). Riga-Fede disease is characterized by traumatic ulceration of the tongue caused by repetitive movement of the tongue over the mandibular incisors (3). Management of Riga-Fede disease may involve smoothing any sharp edges on the lower incisors or extraction of the lower incisors when they are excessively mobile, interfere with comfortable feeding, or when persistent soft tissue trauma is present (3).

While soft tissue ulceration generally resolves after removal of the offending tooth, the development of delayed reactive lesions is unusual in infants (4). Peripheral ossifying fibroma (POF) is a benign reactive gingival lesion common in older children but rarely reported in neonates (1). This report presents a case of POF following natal tooth extraction, underscoring the need for continued monitoring and histopathologic confirmation of infant oral lesions.

## Description of case

A female neonate, one of two monozygotic twins, was born at 36 weeks' gestation. No complications were noted during pregnancy or delivery. At birth, two mandibular central incisor natal teeth were present. The mandibular right primary central incisor exhibited marked mobility and was extracted in the delivery suite. The mandibular left primary central incisor was retained. The twin sibling was not born with natal teeth.

**Figure 1:**
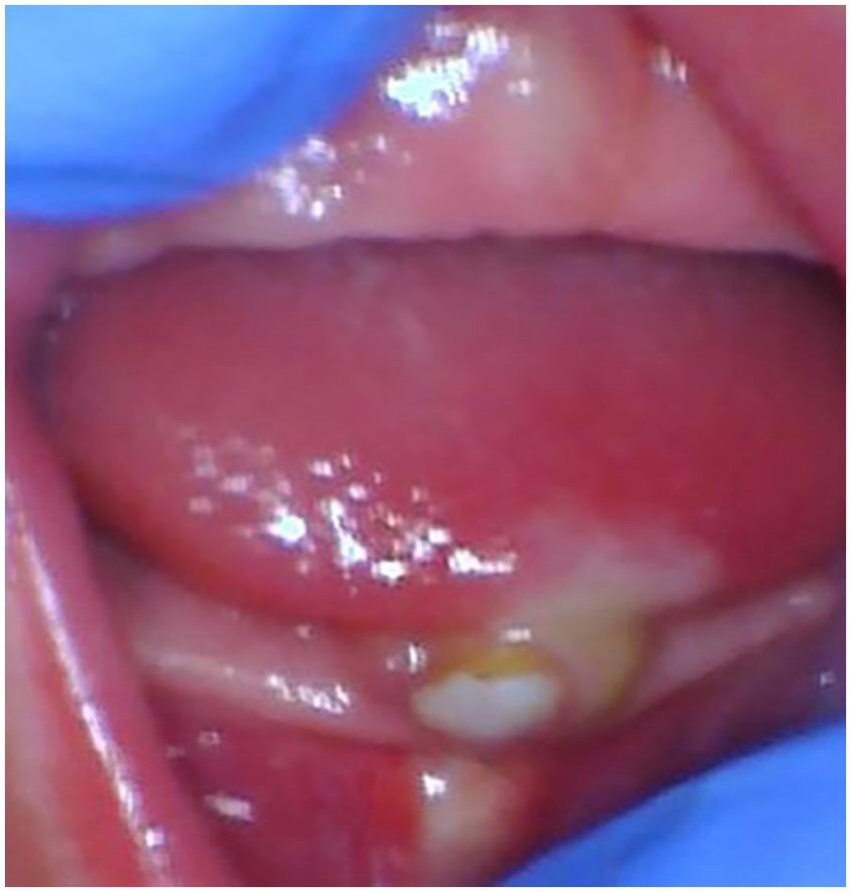


At four days of age, she was referred to a pediatric dental clinic for evaluation of the remaining natal tooth. Clinical examination revealed a Grade III mobile natal mandibular primary central incisor. Adjacent to the tooth, there was a shallow ulcer on the ventral tongue measuring approximately 6 × 4 mm (Figure 1). The gingival mucosa surrounding the natal tooth appeared white-yellow in color with vestibular swelling.

The clinical presentation of the tongue ulcer was consistent with Riga-Fede disease. Due to the mobility of the tooth and the associated aspiration risk, along with trauma to the adjacent tongue mucosa, the remaining natal tooth was extracted. The tooth was removed with forceps in a knee-to-knee position under topical anesthesia.

**Figure 2:**
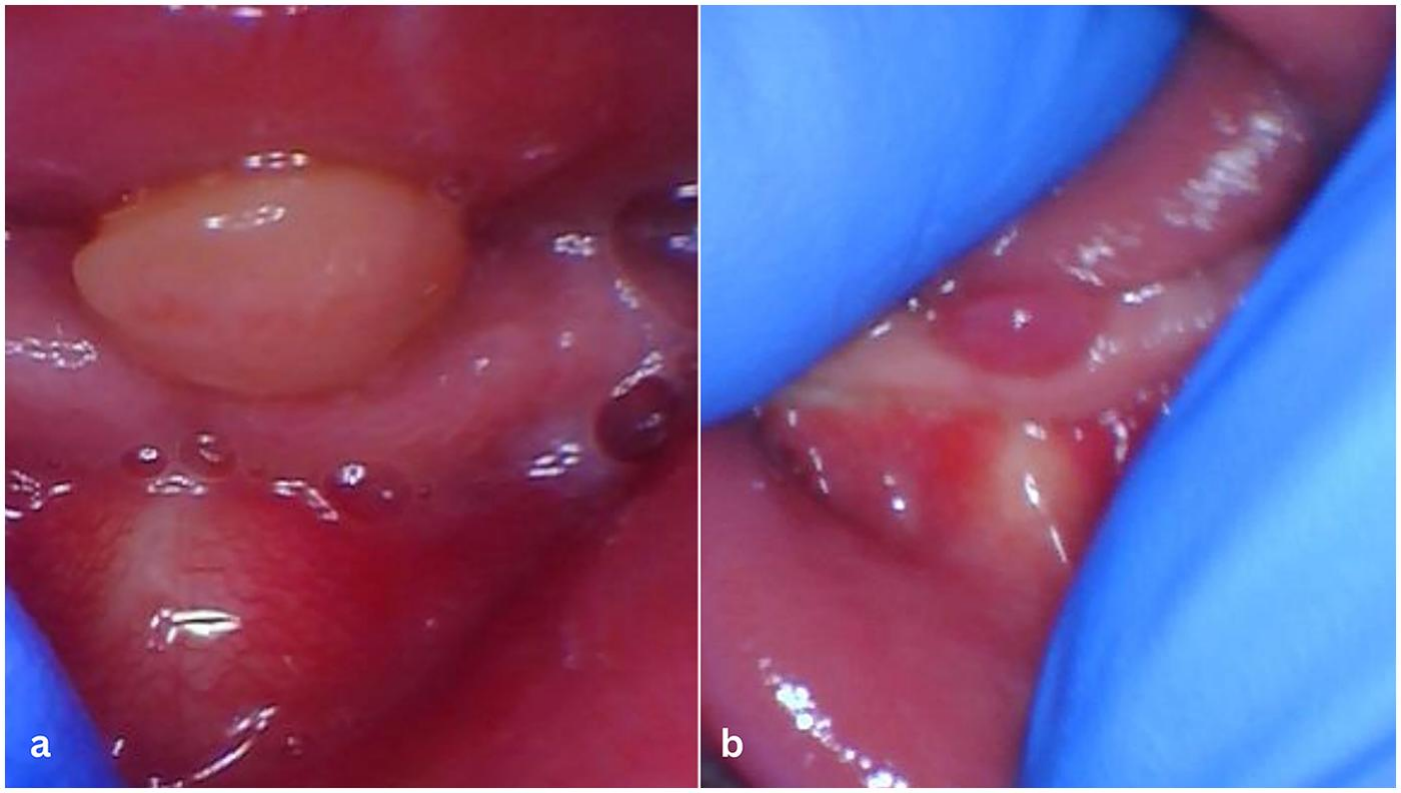


At a two-week post-extraction follow-up, a 4 × 3 mm soft, erythematous gingival swelling was observed at the extraction site (Figure 2). As the lesion was asymptomatic and appeared benign, it was monitored. Over the next several months, the swelling initially appeared to regress (Figure 2b). No bleeding or purulence was noted.

**Figure 3:**
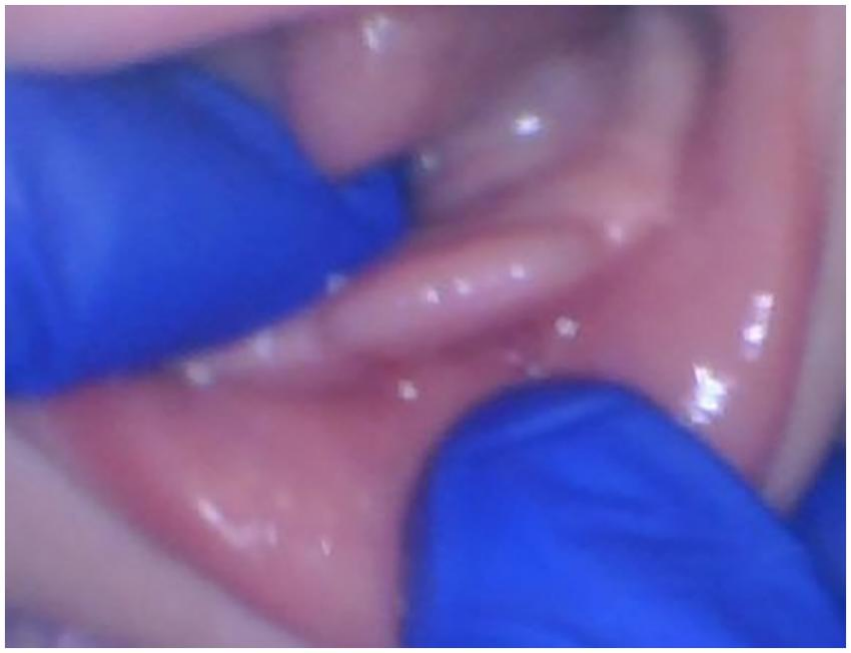


However, at five months post-extraction, the lesion enlarged into a firm, pink, pedunculated mass measuring 15 × 5 × 5 mm (Figure 3). The infant was observed sucking on the lesion, but there was no reported discomfort or feeding difficulty.

Due to the increase in lesion size, an excisional biopsy was performed to confirm a reactive etiology and rule out a neoplastic process. Excision was performed under local anesthesia (2% lidocaine with 1:100,000 epinephrine) using a 940 nm diode laser (Biolase Epic Pro) in continuous mode at 5.6 W with a 400 µm fiber tip and the tissue was fixed in 10% neutral buffered formalin for histopathologic analysis. Figure 3b shows the mandibular anterior biopsy site immediately after excision.

**Figure 4:**
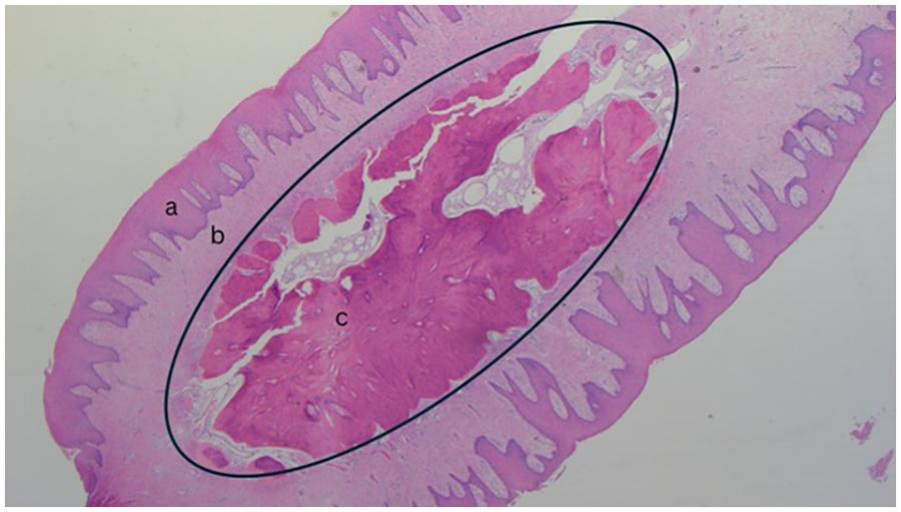


Microscopic examination showed a nodular mass of oral mucosa composed of hyperplastic parakeratinized stratified squamous epithelium overlying a bland fibroblastic proliferation with osteoid, woven, and lamellar bone (Figure 4). The histopathologic findings were consistent with POF.

**Figure 5:**
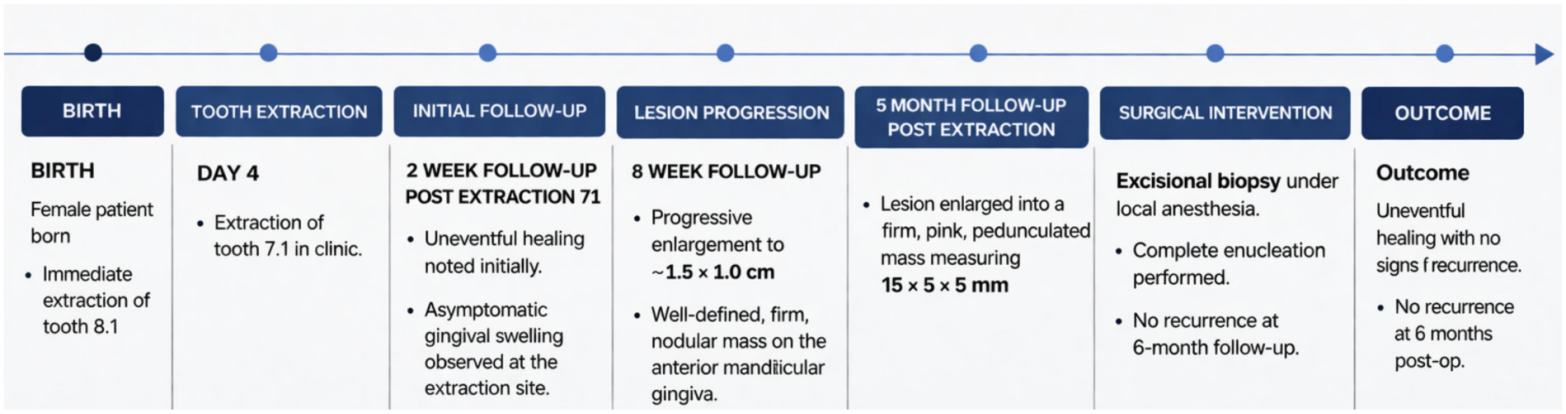
Timeline summarizing the sequence of clinical events from initial presentation to surgical management and follow-up.

At 10-day and 6-month follow-up, the area healed completely with no evidence of recurrence. A structured timeline summarizing the sequence of clinical events from initial presentation to surgical management and follow-up is shown in Figure 5.

## Discussion

POF is a benign reactive gingival lesion accounting for approximately 9%–20% of reactive gingival growths in older children and young adults (4, 5). Its occurrence in neonates is exceedingly rare, with only isolated cases reported in the literature (6, 7). The mandibular anterior region appears to be the most commonly reported site in pediatric cases.

A previously reported case described a clinically similar lesion following natal tooth extraction; however, histopathologic analysis identified it as a pyogenic granuloma rather than POF (8). This distinction underscores the importance of biopsy, as clinically similar lesions may differ significantly in histopathology and recurrence potential.

Reactive oral lesions reported in neonates most commonly include congenital epulis and pyogenic granuloma, which differ in both clinical presentation and timing of onset. Congenital epulis is present at birth and does not develop as a reactive lesion following trauma, whereas pyogenic granuloma typically presents as a rapidly growing, highly vascular lesion with a tendency to bleed. In contrast, the lesion in the present case developed several weeks after natal tooth extraction, initially presenting as a small erythematous swelling that appeared to regress before enlarging into a firm fibrous mass over several months. This delayed and progressive presentation, combined with the histopathologic finding of fibrous connective tissue with calcified material, distinguishes this case from previously reported neonatal reactive lesions and supports the diagnosis of POF.

POF is believed to originate from the periodontal ligament in response to local irritation or trauma, leading to fibroblastic proliferation and subsequent calcified tissue formation. In this case, it is hypothesized that initial chronic trauma from the natal tooth, followed by repetitive mechanical irritation from sucking behavior, may have contributed to lesion development. This proposed mechanism remains speculative but is supported by the established reactive nature of POF and its strong association with local irritants such as trauma, plaque, and calculus (9). Similar reactive gingival lesions have been reported to arise in response to chronic mechanical stimulation, supporting the hypothesis that repetitive sucking behavior in this case may have contributed to lesion development. This aligns with existing literature suggesting that periodontal ligament–derived cells undergo fibroblastic proliferation and subsequent calcification in response to irritation (1, 4, 5).

POF was considered in the differential diagnosis based on the clinical presentation prior to histopathologic confirmation. Clinically, the lesion presented as a localized, firm, pedunculated gingival mass arising from the interdental papilla, which is characteristic of reactive gingival lesions. The differential diagnosis included congenital epulis, pyogenic granuloma, peripheral giant cell granuloma, and fibrous epulis. Pyogenic granuloma was considered due to the patient's age and gingival location; however, the lesion lacked the typical erythematous appearance and bleeding tendency. Peripheral giant cell granuloma was also considered, but there was no bluish-purple discoloration typically associated with that lesion. Fibrous epulis was considered due to the firm consistency; however, the papillary origin and progressive enlargement raised suspicion for a reactive gingival lesion such as POF. Based on these clinical features, POF was considered a provisional diagnosis, which was later confirmed by histopathologic examination demonstrating fibrous connective tissue with calcified material consistent with bone or cementum-like deposits.

In this case, diode laser excision was utilized, offering several advantages, including a bloodless surgical field, reduced operative time, and elimination of the need for sutures. The laser parameters were selected to achieve efficient soft tissue ablation with adequate hemostasis while preserving tissue integrity for histopathologic evaluation. Previous studies have demonstrated that diode laser excision, when properly performed, does not compromise diagnostic accuracy (10).

This case highlights the importance of continued monitoring following natal tooth extraction, as reactive gingival lesions may develop in a delayed manner even after initial healing appears uneventful. Although rare in neonates, POF should be considered in the differential diagnosis of gingival masses arising after extraction or chronic irritation. Early recognition and excisional biopsy with histopathologic evaluation are essential to establish a definitive diagnosis and guide appropriate management.

## Data Availability

The raw data supporting the conclusions of this article will be made available by the authors, without undue reservation.
